# The potential for zoonotic malaria transmission in five areas of Indonesia inhabited by non-human primates

**DOI:** 10.1186/s13071-023-05880-4

**Published:** 2023-08-07

**Authors:** Dendi Hadi Permana, Hasmiwati, Dwi Anita Suryandari, Ismail Ekoprayitno Rozi, Lepa Syahrani, Wuryantari Setiadi, Nuzulia Irawati, Rizaldi, Suradi Wangsamuda, Yenni Yusuf, Irdayanti, Hijral Aswad, Puji Budi Setia Asih, Din Syafruddin

**Affiliations:** 1 Doctoral Program in Biomedical Science, Faculty of Medicine, University of Indonesia, Jakarta, Indonesia; 2 National Research and Innovation Agency (BRIN), Eijkman Research Center for Molecular Biology, Cibinong, Indonesia; 3 Department of Biology, Faculty of Mathematics and Natural Sciences, University of Andalas, Padang, Indonesia; 4 Department of Biology, Faculty of Medicine, University of Indonesia, Depok, Indonesia; 5 Doctoral Program in Faculty of Medicine, University of Hasanuddin, Makassar, Indonesia; 6 Doctoral Program in Department of Biology, Faculty of Mathematics and Natural Sciences, University of Indonesia, Jakarta, Indonesia; 7 Department of Parasitology, Faculty of Medicine, University of Hasanuddin, Makassar, Indonesia

**Keywords:** Zoonotic malaria, Non-human primates, *Plasmodium* species, Infection, *Anopheles*, Malaria vector, Indonesia

## Abstract

**Background:**

Indonesia is home to many species of non-human primates (NHPs). Deforestation, which is still ongoing in Indonesia, has substantially reduced the habitat of NHPs in the republic. This has led to an intensification of interactions between NHPs and humans, which opens up the possibility of pathogen spillover. The aim of the present study was to determine the prevalence of malarial parasite infections in NHPs in five provinces of Indonesia in 2022. Species of the genus *Anopheles* that can potentially transmit malarial pathogens to humans were also investigated.

**Methods:**

An epidemiological survey was conducted by capturing NHPs in traps installed in several localities in the five provinces, including in the surroundings of a wildlife sanctuary. Blood samples were drawn aseptically after the NHPs had been anesthetized; the animals were released after examination. Blood smears were prepared on glass slides, and dried blood spot tests on filter paper. Infections with *Plasmodium* spp. were determined morphologically from the blood smears, which were stained with Giemsa solution, and molecularly through polymerase chain reaction and DNA sequencing using* rplU* oligonucleotides. The NHPs were identified to species level by using the mitochondrial cytochrome *c* oxidase subunit I gene and the internal transcribed spacer 2 gene as barcoding DNA markers. Mosquito surveillance included the collection of larvae from breeding sites and that of adults through the human landing catch (HLC) method together with light traps.

**Results:**

Analysis of the DNA extracted from the dried blood spot tests of the 110 captured NHPs revealed that 50% were positive for *Plasmodium*, namely *Plasmodium cynomolgi*, *Plasmodium coatneyi*, *Plasmodium inui*, *Plasmodium knowlesi* and *Plasmodium* sp. Prevalence determined by microscopic examination of the blood smears was 42%. Species of the primate genus* Macaca* and family Hylobatidae were identified by molecular analysis. The most common mosquito breeding sites were ditches, puddles and natural ponds. Some of the *Anopheles letifer* captured through HLC carried sporozoites of malaria parasites that can cause the disease in primates.

**Conclusions:**

The prevalence of malaria in the NHPs was high. *Anopheles letifer*, a potential vector of zoonotic malaria, was identified following its collection in Central Kalimantan by the HLC method. In sum, the potential for the transmission of zoonotic malaria in several regions of Indonesia is immense.

**Graphical Abstract:**

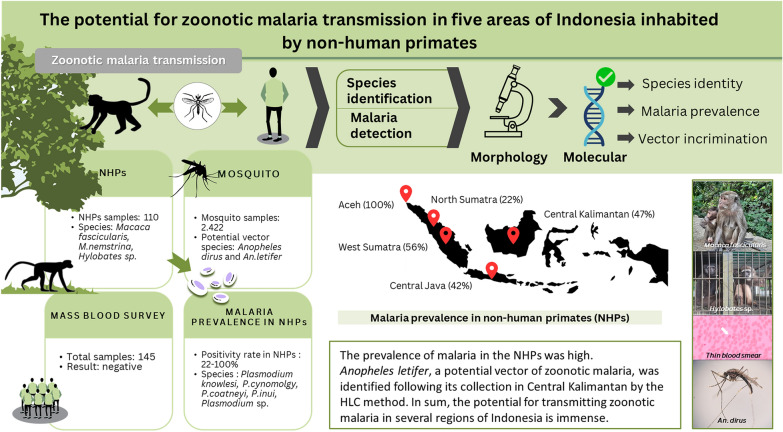

## Background

Malaria, which is one of the oldest infectious diseases, remains a public health problem in Indonesia despite a significant reduction in its incidence in the western parts of the archipelago. Malaria is caused by species of the apicomplexan parasite genus *Plasmodium*, which are transmitted between their vertebrate hosts by mosquito vectors. In Southeast Asia, at least 13 *Plasmodium* species are able to infect non-human primates (NHPs), and seven of them can naturally infect macaques [1–3]. Infection of their natural hosts by any one of these seven *Plasmodium* spp., i.e.,* Plasmodium cynomolgi*,* Plasmodium brasilianum*,* Plasmodium eylesi*,* Plasmodium knowlesi*,* Plasmodium inui*,* Plasmodium schwetzi*, and *Plasmodium simium*, usually results in very low parasitemia and causes mild or asymptomatic disease [4]. *Plasmodium falciparum*, *P. vivax*, *P. malariae*, and *P. ovale* are commonly found in humans, but *P. knowlesi*, which causes zoonotic malaria throughout Southeast Asia, also infects macaques naturally [5, 6]. Thirty-eight species of NHPs have been identified in Indonesia [7–9]; nine of these are macaque species endemic to the country and include the Mentawai pig-tailed macaque (*Macaca pagensis*), the black macaque (*Macaca nigra*), the Moor macaque (*Macaca maura*), Heck’s macaque (*Macaca hecki*), the booted macaque (*Macaca ochreata*), the Tonkean macaque (*Macaca tonkeana*), the Buton macaque (*Macaca brunnescens*), and the Gorontalo macaque (*Macaca nigrescens*) [10, 11]. Is it considered prudent to monitor wild macaques for the *Plasmodium* spp. that they harbor, and the mosquito vectors of these parasites, to better understand the epidemiology of the disease so that feasible, effective measures can be proposed to reduce the risk of zoonotic malaria infection.

Within the last three decades, deforestation for agriculture, mining, and resettlement has substantially reduced the habitat of NHPs in the western part of Indonesia, including the islands of Sumatra, Java, Kalimantan (the Indonesian part of Borneo), Sulawesi, Bali, and the Lesser Sunda Islands [12]. This has led to the intensification of interactions between NHPs and humans in many parts of Indonesia and thus opened up the possibility of pathogen spillover from NHPs, and other wild animals, to humans, as evidenced by reports of cases of zoonotic malaria in Sumatra and Kalimantan [13–15]. The aim of the present study was to determine the prevalence of malarial parasite infections in NHPs in five provinces of Indonesia in 2022. The wild NHPs were captured using traps installed in several localities, including in the surroundings of a wildlife sanctuary that borders settlements, and were released after examination. Settlers who live adjacent to the wildlife sanctuary were investigated for zoonotic malaria infections. Mosquito vectors of malaria were monitored in the five provinces.

## Methods

### Study sites

**Fig. 1:**
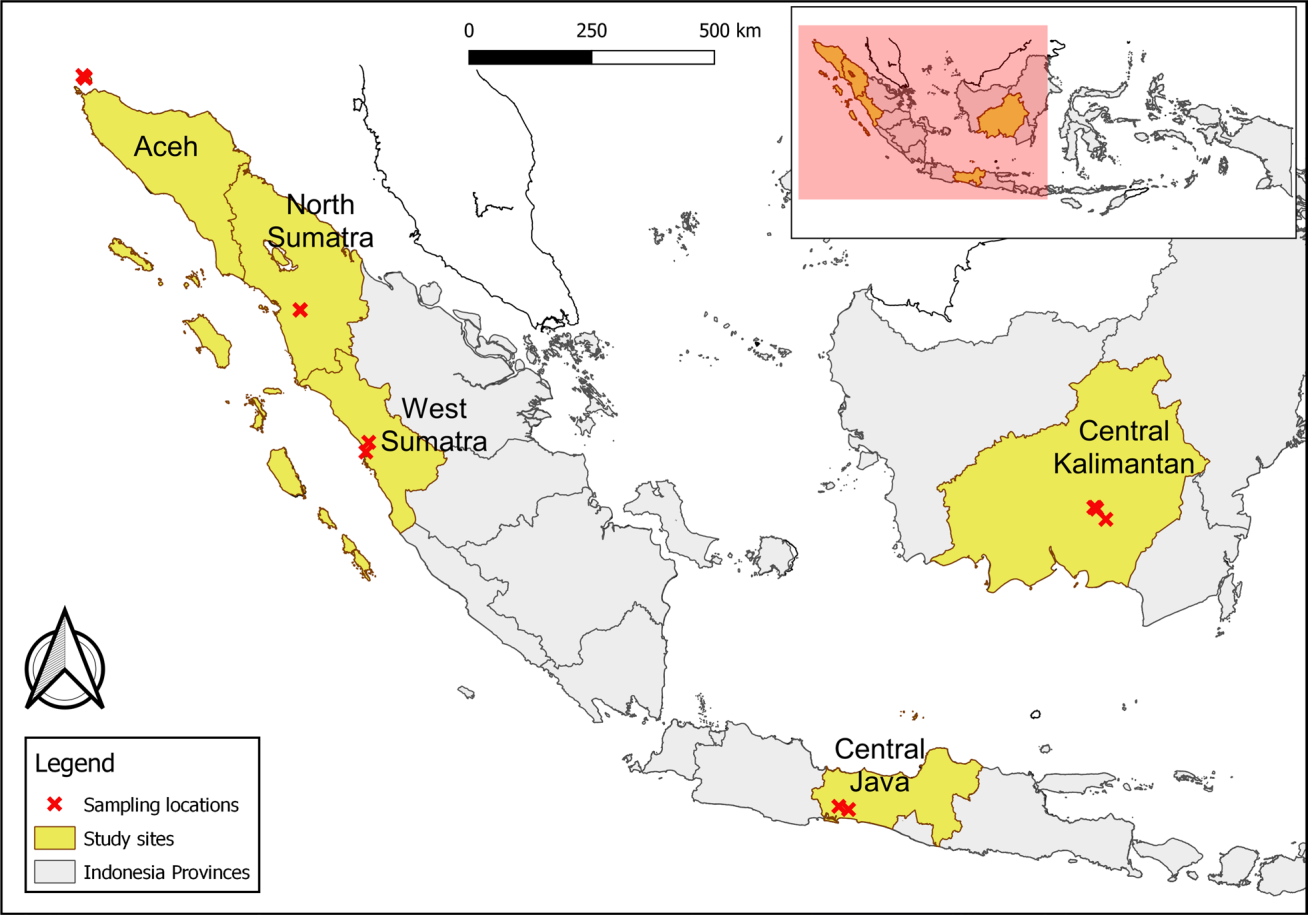
Location of the sampling sites (red crosses) near the boundaries of five provinces of Indonesia

Sampling of NHPs and mosquito vectors was conducted in the following five provinces of Indonesia: Central Java (Cikakak wildlife sanctuary and Kalisalak village in Banyumas Regency); North Sumatra (forest adjacent to Aek Batang Paya village, Sipirok Subdistrict, South Tapanuli Regency); Aceh (Iboih village, Sukakarya Subdistrict, Sabang Municipality; the village is adjacent to Weh Island Natural Park); West Sumatra (Mount Meru, Lubuk Begalung subdistrict and Lubuk Minturun, Koto Tangah district, Padang City Regency); and Central Kalimantan (Nyaru Menteng Arboretum, Taman Wisata Bukit Tangkiling Nature Park, and a settlement near the urban forest of Palangkaraya) (Fig. 1).

### Collection of NHPs

Traps were installed in NHP study sites from August to November 2022 and monitored every day for the presence of trapped NHPs. The NHPs were anesthetized by intramuscular injection with Zoletil (4 mg/kg body weight) (Virbac). Sex, morphological characteristics, weight, and body temperature were recorded for each animal. Blood (3-ml samples) was drawn and then transferred into tubes containing ethylenediaminetetraacetic acid. Thin and thick blood smears were prepared on glass slides, and three drops of blood were collected on Whatman 3 MM filter papers for DNA extraction and molecular analysis.

### Mass blood survey

Mass blood surveys were conducted in Aceh and Central Java. Residents of the village adjacent to the wildlife sanctuary were asked if they would like to voluntarily participate in the study after they had received information about it. Blood was obtained from the participants by aseptic finger prick and the drops collected on Whatman filter paper for dried blood spot testing and on a glass slide for the preparation of thin and thick blood smears, in accordance with standard procedures of the World Health Organization [16].

### Microscopic analysis

Thick and thin blood films of the NHPs and human participants were stained with Giemsa and examined under light microscopy using a 100x objective lens with immersion oil for the presence of malaria parasites. At least 200 microscopic fields of the thick smear were examined to determine parasite densities; if no parasites were found the sample was considered negative.

### Mosquito breeding site survey

Water bodies in the study areas were surveyed for the presence of mosquito larvae using a standardized World Health Organization protocol [17]. Larvae were recorded at various sampling locations to identify and evaluate breeding habitats. The different types of water bodies were characterized according to depth, water clarity, and vegetation, and the geographic coordinates recorded using the Open Data Kit (ODK) Collect application. The mosquito larvae in each water body were counted and collected using a dipper and pipette. The salinity and pH of the water were recorded during sampling.

### Collection of adult mosquitoes and DNA extraction

Mosquito species diversity was determined using the human landing catch (HLC) method. Residents that voluntarily participated in the study were trained in the HLC method. Two collectors were assigned per house, one positioned indoors and one outdoors (on the veranda). The volunteers collected the mosquitoes that landed on their exposed lower legs by using a mouth aspirator. Collections were conducted for 50 min every hour from 1800 to 0600 hours. The mosquitoes were immediately killed in the field with chloroform vapor and transported in a tube containing silica gel. The adult mosquitoes were identified to species based on their morphology by the use of illustrated keys [18]. A randomly selected subset of morphologically identified samples was also molecularly identified by sequencing the internal transcribed spacer 2 (*ITS2*) and cytochrome oxidase subunit I (*COI*) genes [19–23]. These samples were chosen to represent all of the morphologically identified specimens and the locations in which they were collected. The mosquito samples were ground with a Teflon pestle and then transferred to 1.5-ml Eppendorf microtubes containing 50 µl of water and 100 µl saponin. Mosquito DNA was extracted using a Chelex-100 ion exchanger (Bio-Rad Laboratories, Hercules, CA) according to a published procedure [24]. The DNA was either used immediately for polymerase chain reaction (PCR) or stored at -20 °C for later analysis.

### DNA extraction for NHPs and parasites

Parasite DNA and NHP DNA was extracted from the NHP blood samples using a QIAamp DNA Mini Kit (QIAGEN, Hilden, Germany). The DNA was either used immediately for PCR amplification or stored at − 20 °C for later analysis.

### Molecular analyses

Extracted genomic DNA was amplified by PCR targeting the* ITS2* and* COI* genes for species identification of the mosquitoes, and only *COI* for the NHP samples. The forward and reverse gene-specific oligonucleotides for *ITS2* were ITS2A (5ʹ-TGTGAACTGCAGGACACAT-3ʹ) and ITS2B (5ʹ-TATGCTTAAATTCAGGGGGT-3ʹ) [24], respectively; the universal primers used for *COI* were LCO1490 (5'-GGTCAACAAATCATAAAGATATTGG-3') and HCO2198 (5'-TAAACTTCAGGGTGACCAAAA AATCA-3ʹ) [25]. The PCR conditions for* COI* amplification were as follows: incubation at 95 °C for 1 min, followed by 35 cycles at 95 °C for 15 s, 53 °C for 15 s, and 72 °C for 60 s, with final extension at 72 °C for 5 min. For *Plasmodium* sp. detection, a semi-nested PCR assay was used targeting the small subunit ribosomal RNA genes, using primers rplU1 (5ʹ-TCAAAGATTAAGCCATGCAAGTGA-3ʹ) and rplU5 (5ʹ-CCTGTTGTTGCCTTAAACTCC-3ʹ), and a second PCR using rplU1 and rplU4 (5ʹ-TACCCGTCATAGCCATGTTAGGCCAATACC-3ʹ) [22]. The PCR conditions were as described in [26]. All the amplicons were visualized by using gel electrophoresis with 1–2% agarose gels. The consensus sequences of these small subunit ribosomal RNA genes were compared to those in the NCBI nr database by using BLAST to confirm species identification.

## Results

In the North Sumatra site, the NHP habitat partly comprised a plantation. In the other sites, the NHPs lived in their natural habitat but also interacted with settlers in the surroundings. A total of 110 NHPs were captured during a 20-day period in the traps that had been installed in the five study areas. The baseline characteristics of the NHPs are shown in Table 1. Sixty-one of the NHPs were male and 49 were female. Body weight of the NHPs ranged from 3.4 to 5.5 kg, and the average body temperature from 36.7 to 39.5 °C. The NHPs were either macaques (family Cercopithecidae) and gibbon (family Hylobatidae). Most of the macaques that were trapped were long-tailed macaques, and a few were pig-tailed macaques. The distributions of the NHPs sampled in this study are summarized in Table 2.

**Table 1:** Baseline characteristics (sex, average body temperature, and average body weight) of endemic non-human primates (NHPs) captured in five provinces of Indonesia

Study site	Male	Female	Average body temperature (°C)	Average body weight (kg)
Central Java	18	18	36.7	5.5
North Sumatra	5	4	39.0	4.9
West Sumatra	14	13	ND	ND
Aceh	14	3	39.5	5.0
Central Kalimantan	10	11	38.4	3.4

*ND* Not determined

**Table 2:** Morphological and molecular (polymerase chain reacion; PCR) identification of endemic NHPs captured in five provinces of Indonesia

Site	Type of identification													
	Morphological				Molecular^a^									
	*Macaca fascicularis*	*Macaca nemestrina*	*Hylobates* sp.	*n*	*Hoolock hoolock*^b^	*Hylobates albibarbis*	*Macaca arctoides*^b^	Macaca brunnescens*	*M. fascicularis*	*Macaca mulatta*^b^	*M. nemestrina*^b^		Unidentified^c^c	*n*
Central Java	36	–	–	36	–	–	25	–	–	5	4		2	36
North Sumatra	5	4	–	9	–	–	1	–	–	6	–		2	9
West Sumatra	26	1	–	27	–	–	4	1	1	10	4		7	27
Aceh	17	–	–	17	–	–	9	–	–	–	3		5	17
Central Kalimantan	16	3	2	21	1	1	7	–	–	8	3		1	21

^a^Species identification confirmed by PCR with mitochondrial DNA markers

^b^Species similarity was ≤ 98%; for the other NHPs, species similarity was > 98%

^c^Sequencing unsuccessful

### Molecular identification of the NHP species

PCR amplification of the *COI* gene was successful for 93 of the captured NHPs and unsuccessful for 17 of them. Amplicon sequencing confirmed that these 93 NHPs included individuals of the two species of macaque identified morphologically, namely *Macaca fascicularis* and *Macaca nemestrina*. Two species of the family Hylobatidae were also identified molecularly, *Hoolock hoolock* and *Hylobates albibarbis*. Variation in the DNA sequence of the *COI* gene of *M. fascicularis* was noted between some of the study sites.

### Prevalence of *Plasmodium* spp. infections in NHPs

**Fig. 2:**
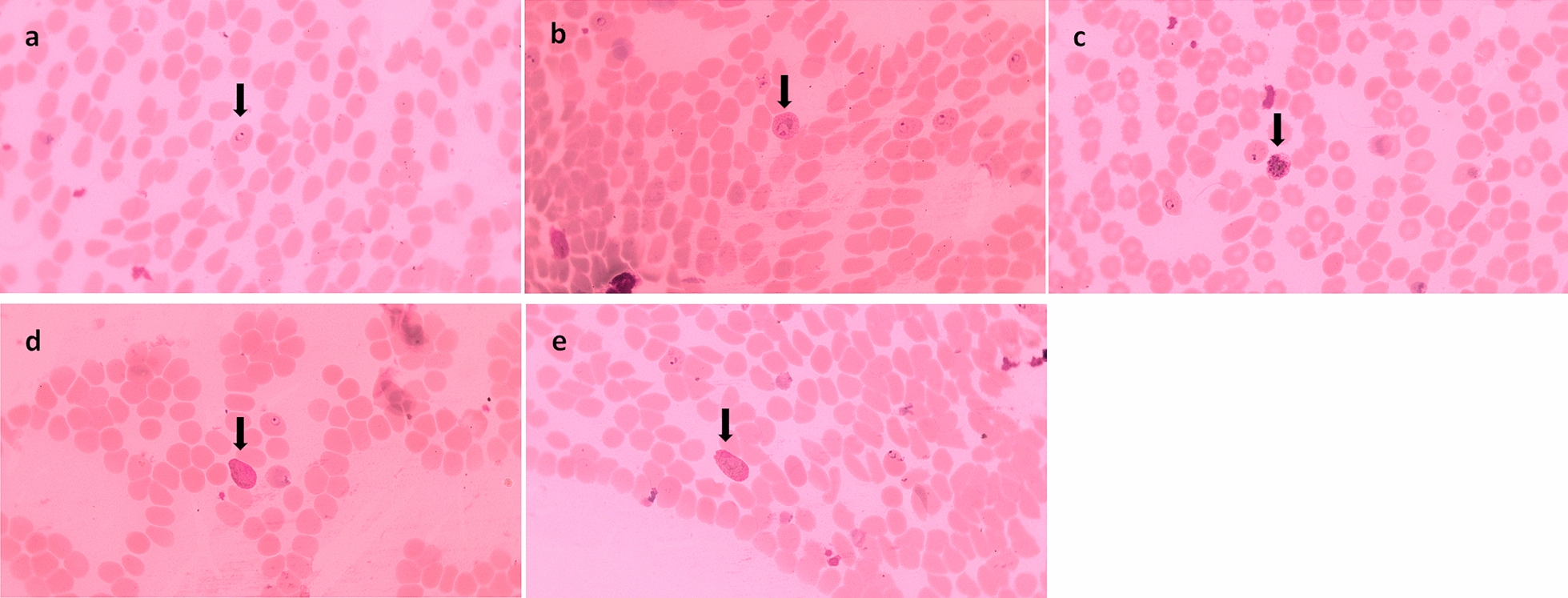
*Plasmodium cynomolgi*; **a** ring form; **b** trophozoite; **c** merozoite; **d** male gametocyte; **e** female gametocyte

**Fig. 3:**
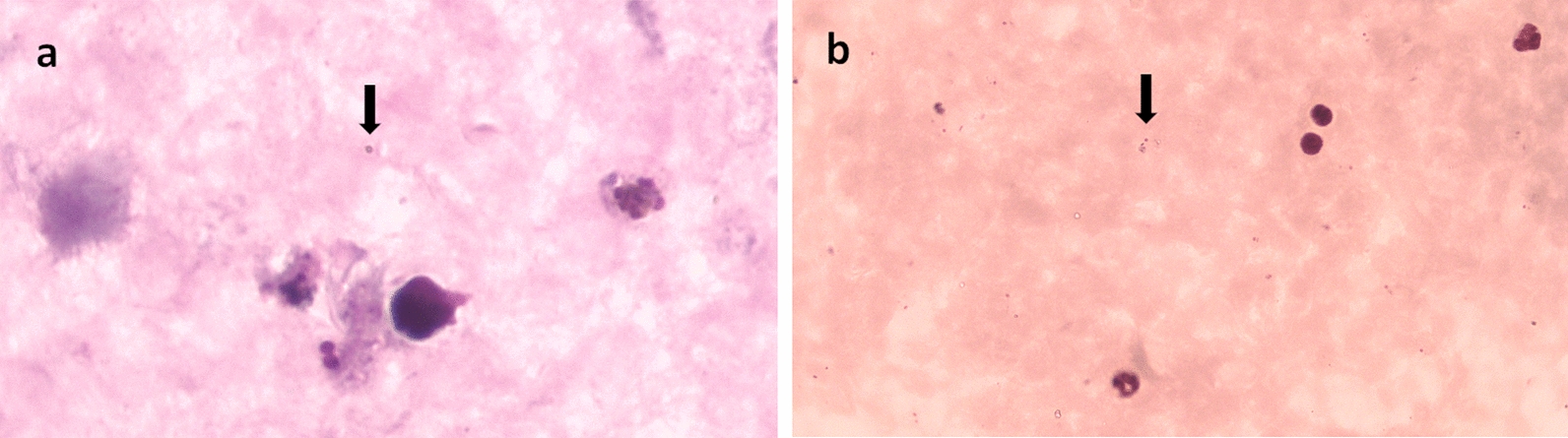
*Plasmodium coatneyi* and *Plasmodium knowlesi*; **a** ring form of *P. coatneyi*; **b** ring form of *P. knowlesi*

**Fig. 4:**
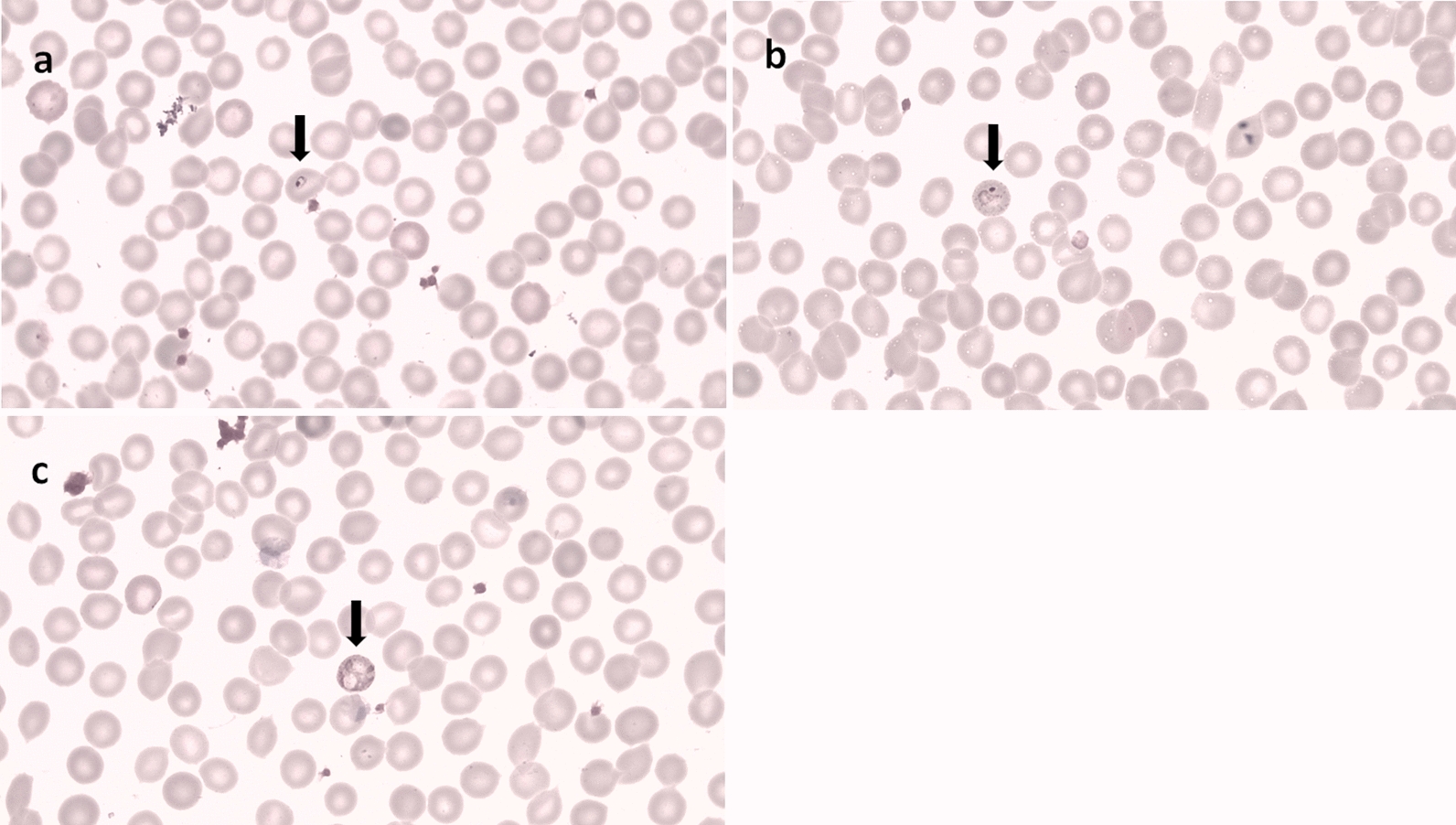
*Plasmodium inui*; **a** ring form; **b** trophozoite stage; **c** merozoite stage

Of the total 110 NHPs captured in the five provinces, *Plasmodium* infections were morphologically diagnosed in 47 individuals (Table 3). However, molecular analysis using the ribosomal DNA or mitochondrial *COI* genes indicated that 55 of the NHPs were infected with *Plasmodium* spp. (Table 3), namely *Plasmodium inui*, *Plasmodium cynomolgi*, *Plasmodium coatneyi*, *Plasmodium knowlesi*, and other *Plasmodium* spp. (Table 4; Figs. 2, 3, 4). The highest rate of *Plasmodium* infection in the macaques was in Iboih village, Aceh. No *P. knowlesi* infection was found among the macaques captured in Aceh or North Sumatra. The most prevalent species were *P. inui* and *P. cynomolgi*, followed by *P. coatneyi* and then *P. knowlesi* (Table 4). *Plasmodium* sp. was identified in 16 of the NHP positive for malaria. Two species of the genus* Hylobates* identified in this study were not infected with any species of *Plasmodium*.

**Table 3:** The number of individual NHPs diagnosed with malaria in the five study areas

	Method of malaria diagnosis			
	Microscopy		PCR	
	Positive (no. individual NHPs)	Negative (no. individual NHPs)	Positive (no. individual NHPs)	Negative (no. individual NHPs)
Central Java	15	21	12	24
North Sumatra	2	7	2	7
West Sumatra	15	12	15	12
Aceh	15	2	17	-
Central Kalimantan	–	21	9	12
Total	47	63	55	55

**Table 4:** *Plasmodium* species identified in endemic malaria-positive NHPs from the five provinces

Location	*Plasmodium coatneyi*	*Plasmodium cynomolgi*	*Plasmodium inui*	*Plasmodium knowlesi*	*Plasmodium* sp.	Total
Central Java	–	–	2	2	8	12
North Sumatra	–	–	–	–	2	2
West Sumatra	–	1	9	5	–	15
Aceh	1	9	7	–	–	17
Central Kalimantan	–	–	1	2	6	9
Total	1	10	19	9	16	55

### Morphology of the primate malaria species

Figures 2, 3 and 4 show morphological characteristics of different stages of the *Plasmodium* species found in this study. Several blood stages of *P. cynomolgi* are shown in Fig. 2. The infected red blood cells are distinctly enlarged. *Plasmodium coatneyi* and *P. knowlesi* are difficult to distinguish morphologically in thick and thin blood smears. *Plasmodium inui*, which can cause malaria in NHPs, clearly shows a pigmented trophozoite stage in the red blood cells.

### Mosquito breeding sites

**Fig. 5:**
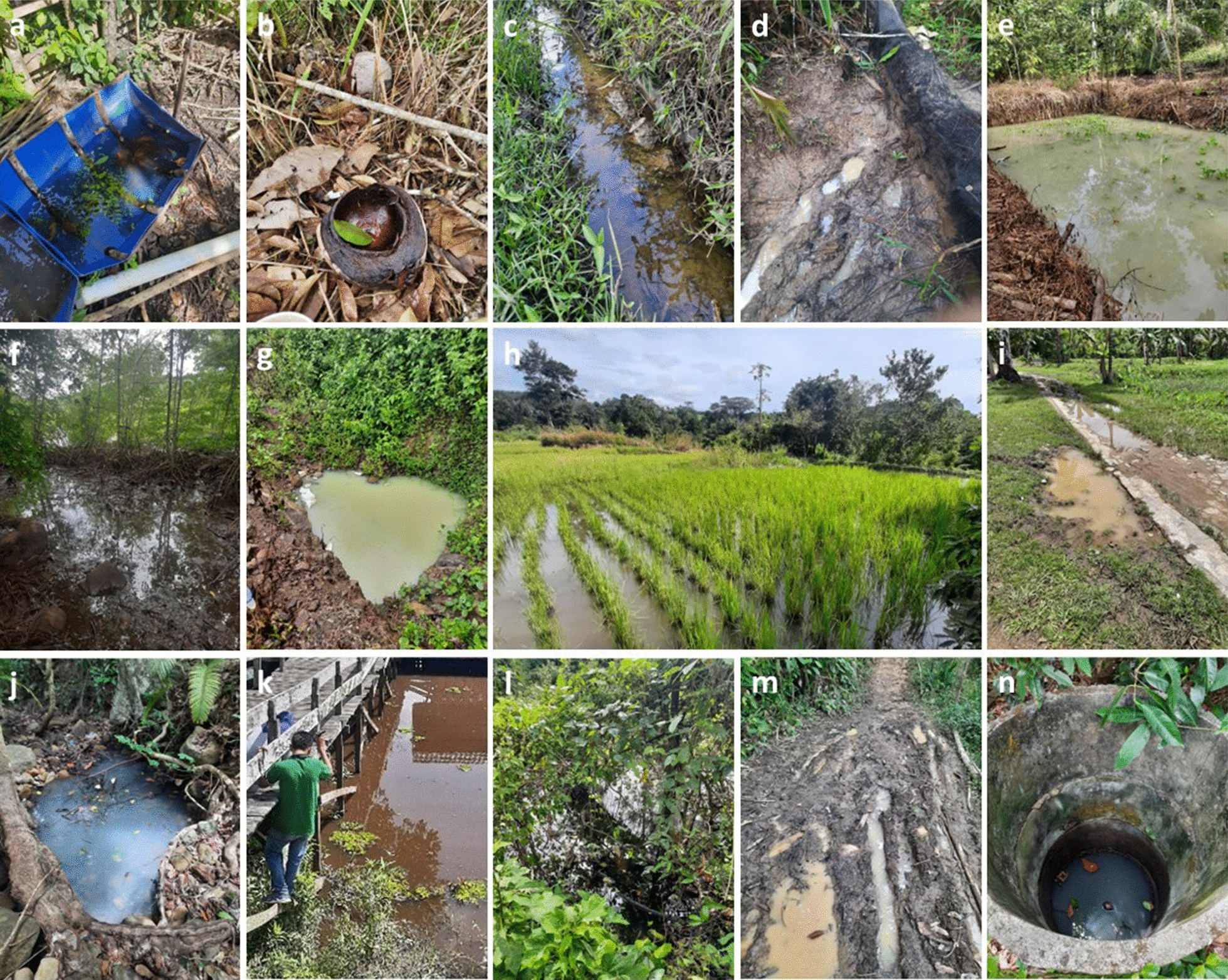
Types of mosquito breeding sites. **a** Artificial water container; **b** coconut shell, leaf, tree hole; **c** ditch; **d** hoof print; **e** man-made pond; **f** mangrove; **g** natural pond; **h** paddy field; **i** puddle; **j** spring; **k** stream margin; **l** swamp; **m** tire track; **n** well

Fourteen types of mosquito breeding sites were found (Table 5; Fig. 5), of which ditches and puddles were the most common. The types of mosquito breeding sites that contained *Anopheles* larvae included artificial water containers, coconut shells, ditches, hoof prints, man-made ponds, mangroves, natural ponds, paddy fields, puddles, springs, stream margins, swamps, tire tracks, and wells. The pH of the breeding sites ranged from 2.5 to 9.6, and the salinity from 0 to 23 p.p.m.

**Table 5:** Breeding sites of *Anopheles* spp. larvae in the five study areas

Anopheline larval habitats	Percentages of sampled habitat types positive for larvae^a^				
	Central Java	North Sumatra	West Sumatra	Aceh	Central Kalimantan
Artificial water container	− (3)	− (1)	–	− (2)	− (2)
Coconut shell, leaf, tree hole	− (2)	− (1)	–	− (1)	–
Ditch	38 (8)	− (5)	–	20 (5)	21 (14)
Hoof print	–	–	–	100 (1)	–
Man-made pond	50 (2)	–	100 (1)	− (2)	–
Mangrove	–	–	–	− (1)	–
Natural pond	67 (9)	60 (5)	–	17 (6)	50 (2)
Paddy field	67 (6)	60 (5)	–	-	–
Puddle	100 (1)	22 (9)	–	− (11)	18 (11)
Spring	100 (1)	− (1)	–	–	–
Stream margin	− (3)	− (1)	–	–	− (2)
Swamp	–	–	–	–	100 (1)
Tire track	100 (1)	33 (3)	–	–	100 (1)
Well	–	–	–	(1)	–

^a^The number of each habitat type sampled is given in parentheses

### Collection of adult mosquitoes

Species of the genus *Anopheles* and other species of mosquito were collected in Central Java, North Sumatra, Aceh, and Central Kalimantan (Table 6). Mosquito collection was limited to larvae only in West Sumatra due to time constraints. Many species of mosquito in addition to *Anopheles* spp. were collected by HLC. Mosquito diversity was highest in Central Kalimantan, where the predominant species collected was *An. letifer*, and lowest in Central Java. The predominant species collected in North Sumatra was *Anopheles kochi* and in Aceh *Anopheles dirus*.

**Table 6:** Number of adult mosquitoes of each species collected using the human landing catch method in four of the five provinces where the NHPs are endemic

Species		Central Java	North Sumatra	Aceh	Central Kalimantan	Total
*Anopheles*						
*Anopheles dirus*		–	–	9	–	9
*Anopheles kochi*		–	181	–	–	184
*Anopheles montanus*		–	–	1	–	1
*Anopheles nigerrimus*		–	–	3	–	3
*Anopheles letifer*		–	–	–	119	119
*Anopheles umbrosus*		–	–	–	4	4
Total						414
Other species of mosquito						
*Aedes* sp.		1		18	43	62
*Armigeres* sp.		19	2	14	92	127
*Coquillettidia* sp.		1	–	–	2	3
*Culex* sp.		7	618	343	190	1158
*Mansonia* sp.		1	–	–	641	642
Unidentified sp.		–	–	–	16	16
Total						2008

Adult mosquitoes were not collected in West Sumatra

### Morphological and molecular identification of the mosquitoes

Based on the morphology of the adult mosquitoes, six species of *Anopheles* could be identified: *Anopheles dirus*,* Anopheles kochi*, *Anopheles montanus*, *Anopheles nigerrimus*, *Anopheles letifer*, and *Anopheles umbrosus* (Table 7). The other identified mosquito species were *Armigeres subalbatus*, *Culex quinquefasciatus*, *Culex vishnui* and *Mansonia* sp. The molecular analysis confirmed the presence of* An. dirus*,* An. kochi*,* An. sinensis*,* An. sundaicus* and *An. vagus*.

**Table 7:** Results of the molecular identification, using internal transcribed spacer II gene sequencing internal transcribed spacer II gene sequencing, to species level of adult mosquitoes collected by HLC in four of five provinces endemic for the NHPs

Species	Central Java	North Sumatra	West Sumatra	Aceh	Central Kalimantan	Total
*Aedes albopictus*	–	–	–	5	–	5
*Anopheles dirus*	–	–	–	12	–	12
*Anopheles kochi*	–	23	3	-	–	26
*Anopheles sinensis*	–	–	–	2	–	2
*Anopheles sundaicus *sensu lato	–	–	–	2	–	2
*Anopheles vagus*	–	–	21	-	–	21
*Armigeres subalbatus*	–	–	–	2	–	2
*Culex quinquefasciatus*	–	–	–	1	–	1
*Culex vishnui*	–	–	–	1	–	1
*Mansonia* sp.	–	–	–	–	1	1
Total	0	23	24	25	1	73

### Vector incrimination for zoonotic malaria

The PCR amplification of samples from 399 adult *Anopheles* collected through HLC revealed that two were positive for *Plasmodium* DNA. These positive samples were identified as *An. letifer*, captured in the Nyaru Menteng Arboretum in Central Kalimantan (Table 8).

**Table 8:** Entomological indices

Species	*n*	HBR	MHD	Sporozoite rate	EIR
*Anopheles dirus*	9	0.31	0.03	–	–
*Anopheles kochi*	181	7.54	0.75	–	–
*Anopheles montanus*	1	0.03	0.00	–	–
*Anopheles nigerrimus*	3	0.10	0.01	–	–
*Anopheles letifer*	199	6.22	0.62	1.01%	0.06
*Anopheles umbrosus*	5	0.16	0.02	–	–
*Anopheles tessellatus*	1	0.03	0.00	–	–

*HBR* Human biting rate (bites per person per night), *MHD* man-hour density*, EIR* entomological inoculation rate (HBR × sporozoite infection rate)

### Mass blood survey

Limited mass blood surveys for zoonotic malaria infection were conducted in areas of Aceh and Central Java where there is intense interaction between human and NHPs. In Aceh, 36 residents of Iboih village participated in the blood survey; in Central Java, 123 residents participated in the survey. No cases of zoonotic malaria were found in either study area.

## Discussion

There was a high prevalence of *Plasmodium* infection within the macaque populations examined in the five provinces, with the highest prevalence in Sabang, Aceh. The most common *Plasmodium* species identified in the five provinces were *P. inui* and *P. cynomolgi*. *Plasmodium knowlesi*, the most common cause of zoonotic malaria, was only found in West Sumatra, Central Kalimantan, and Central Java. Our findings are not consistent with reports of zoonotic malaria in Acen [15]. In Sabang, Aceh, cases of zoonotic malaria were reported to have been caused by *P. knowlesi* [15, 27], but we found no *P. knowlesi* infection among the macaques examined in the present study. In addition, *Plasmodium cynomolgi*, *Plasmodium inui*, and *Plasmodium fieldi* were reported to have caused zoonotic malaria in NHP in South Sumatra, Bintan Island, West Java, and Lampung [28–30]. The discrepancies between these and our findings indicate the importance of regular monitoring for the detection of zoonotic malaria in these areas and the implementation of mitigation efforts to prevent its spread. The lowest prevalence, which was found in North Sumatra, may be attributable to the fact that fewer NHPs were captured there. It is particularly noteworthy that although human–macaque interactions are relatively intense on the fringes of Cikakak sanctuary, Central Java, no cases of zoonotic malaria cases have ever been reported in the province. This is probably due to the absence from this region of *Anopheles* vectors that are capable of transmitting primate malaria to humans. Vectors of zoonotic malaria thus far identified are mainly of the*Anopheles leucosphyrus* group, with a few other species, such as *Anopheles kochi* and *Anopheles letifer*, also capable of transmitting the disease [31–35].

*Macaca fascicularis* was the most common NHP species trapped in the study areas, and the molecular analysis revealed high DNA sequence variation between individuals from different study sites. This latter finding corroborates the identification of different subspecies of *M. fascicularis*, from Sumatra, Kalimantan, Java, Bali, Sumba, and Timor [10]. *Macaca fascicularis* live in primary and secondary forest in lowland areas and in highland areas at > 1000 m above sea level. In highland areas, they are typically found in secondary forest or agricultural areas [10]. In the present study, most of the *M. fascicularis* were trapped in Central Java, West Sumatra and Aceh, which may be explained by the fact that domesticated, captive macaques predominate in North Sumatra and Central Kalimantan. This, in turn, may explain the absence or lower prevalences of *Plasmodium* spp. infection in these latter two areas. Based on information from villagers in North Sumatra and Central Java, *M. fascicularis* often scavenge for food in settlements. NHPs in areas popular with tourists in Iboih, Aceh, and Cikakak, Central Java are often fed by the visitors, which causing changes in the natural behavior of these animals and encourages them to spend more time around settlements in search of food. Environmental changes such as deforestation and urban expansion are also linked to an increase in human-macaque interactions, which increase the potential for zoonotic transmission of malaria [36].

Several *Anopheles* species, including *An. dirus* collected in Sabang and *An. kochi* collected in North Sumatra, were identified following their capture through HLC, but only *An. letifer*, caught in Central Kalimantan, was infected with *Plasmodium* sp. The DNA of this parasite was found in the head-thoracal parts of the mosquito, which indicated the presence of sporozoites. It is highly likely that *An. dirus* plays an important role in zoonotic malaria transmission in Sabang, the area in which it was found in the present study, as it has been reported to transmit the disease in many other sites in Southeast Asia [37]. Further surveillance is required to elucidate the role of *An. kochi* in the transmission of zoonotic malaria in North Sumatra. In Central Kalimantan, and particularly in Nyaru Menteng Arboretum, where orangutans (*Pongo pygmaeus*) and macaques are protected, *An. letifer*, which bites the NHPs and settlers that live in the area, has been confirmed as a zoonotic malaria vector. The role of *An. letifer* as a vector of zoonotic malaria has also been described for Sarawak [38]. The very low number of individuals of* Anopheles* species sampled during the present study, with the exception of *An. kochi*, may partly explain the low sporozoite rates reported here.

The intensity of NHP–human interactions in the five areas surveyed in this study indicate the potential for zoonotic malaria transmission where the mosquito vectors of this disease are present. At present, mosquito vector surveillance and mass blood surveys of the settlers who live adjacent to the NHPs in these areas are inadequate. We propose that regular vector surveillance should be mandatory in these areas, as should mass blood surveys, particularly for the settlers who live around the forest habitats of the NHPs and the visitors to them, as they are the groups most at risk of zoonotic malaria infection.

## Conclusions

The increased potential for spillover infection of malaria carried by NHPs due to the ongoing reduction in their habitat and consequent increase in their interactions with humans should be monitored by regular vector surveillance and mass blood surveys. A further study is underway in four localities in Indonesia to determine the factors that may contribute to the transmission of zoonotic malaria.

## Data Availability

All of the datasets generated and analyzed during this study are included in the manuscript.
